# The potential of antimicrobial peptides to treat oral infections and cancer

**DOI:** 10.3389/fmed.2025.1712514

**Published:** 2026-01-06

**Authors:** Ana Emilia Carvalho de Paula, Carla Silva Siqueira, Esteban Nicolás Lorenzón

**Affiliations:** 1 Institute of Health Sciences, Federal University of Jataí, Jataí, Goiás, Brazil; 2 Odontology Faculty, Federal University of Uberlândia, Uberlândia, Minas Gerais, Brazil

**Keywords:** antimicrobial peptides, oral infection, oral cancer, antitumoral peptides, drug resistance

## Abstract

Oral cancer represents a significant cause of mortality and morbidity, especially when discovered late. Microbial infections, particularly those caused by *Porphyromonas gingivalis* and *Fusobacterium nucleatum*, play a crucial role in cancer development and prognosis, especially in the oral cavity. Unfortunately, the current pharmacological arsenal for treating infections and oral cancer has a low therapeutic spectrum and high levels of resistance. In this regard, some antimicrobial peptides (AMPs) appear to be potential therapeutic agents, as they exhibit direct cytotoxic activity against bacterial and cancer cells with a low propensity for resistance. This study aimed to review the current literature on dual-function peptide molecules with the potential to treat oral infections and cancer. Peptides such as Nal-P113, hCAP_(109–135)_, and Nisin Z exhibit both antimicrobial and antitumor activity, making them promising therapeutic agents for the prevention and treatment of oral infections and cancer. However, owing to the limitations of AMPs, further *in vitro* and *in vivo* safety and efficacy studies are needed before their commercialization.

## 1 Introduction

### 1.1 Oral cancer

Head and neck cancers (HNC) comprise malignant neoplasms originating from squamous epithelial cells located in the mucosal lining of the upper aerodigestive tract (1). The World Health Organization identifies oral and oropharyngeal carcinomas as among the most prevalent malignancies within this anatomical region (2). Although considerable progress has been achieved in both diagnostic and therapeutic modalities, oral cancer continues to represent a substantial global burden in terms of incidence and mortality. Alarmingly, projections indicate a 65% increase in its occurrence by the year 2050 (3).

Surgical resection is often the primary treatment for oral cancer, while chemotherapy, radiation, and immunotherapy are employed individually or in combination for advanced or inoperable cases. However, the choice of optimal treatment remains controversial (4). Standard chemotherapy protocols for oral cancer frequently involve the use of cisplatin, 5-fluorouracil, and paclitaxel, administered either as single agents or in combination (5). Nevertheless, tumors that initially respond to these drugs often develop resistance, reducing their effectiveness and resulting in poor prognosis. Resistance to chemotherapy can develop through intrinsic or acquired mechanisms. The principal mechanisms of resistance involve decreased drug uptake and increased efflux, enhanced DNA repair capacity, inhibition of drug activation, receptor modifications, and attenuation of drug-induced cellular damage (6, 7). In addition, epigenetic alterations and interactions within the tumor microenvironment play significant roles in the development of chemoresistance.

### 1.2 Oral infections

The tumor microenvironment has also been recognized as a pivotal contributor to the process of carcinogenesis. Increasing scientific evidence suggests that infections caused by bacteria, viruses, fungi, and parasites—especially those linked to chronic infection—are significantly involved in cancer development (8).

Among viruses, human papillomavirus, particularly types 16 and 18, is strongly associated with oropharyngeal squamous cell carcinoma (9), while Epstein–Barr virus plays a key role in the pathogenesis of nasopharyngeal carcinoma (10). Also, Fungal organisms found in the oral cavity, such as *Candida albicans*, can promote carcinogenesis (11). In addition, certain oral parasites have been investigated for their potential oncogenic effects; for example, *Entamoeba gingivalis*, frequently identified in periodontal pockets, has been suggested to enhance tissue destruction and inflammatory responses that may support a pro-tumorigenic environment (12), while Trichomonas tenax has been linked to chronic periodontal inflammation that might indirectly contribute to carcinogenic processes (13).

Infectious agents are a significant cause of cancer, especially in developing countries where resources to control the disease are limited (14). These pathogens may contribute to cancer development through several mechanisms, including the induction of chronic inflammation, genomic instability via host cell DNA damage, immune system suppression, promotion of uncontrolled cellular proliferation, and alterations to the tumor microenvironment (15). Among these infectious agents, there has been a growing focus on bacteria linked to oral diseases, which have increasingly been connected to head and neck cancers. Bacterial involvement in these tumors may arise from disruption in the oral microbiota. Specific bacterial species associated with gingivitis, periodontitis, dental caries, and endodontic abscesses can instigate both acute and chronic infections in the oral cavity, resulting in significant tissue changes (16). *Porphyromonas gingivalis* and *Fusobacterium nucleatum*, two key pathogens in oral diseases, have demonstrated the capacity to induce inflammatory cytokine production and promote cellular proliferation through various mechanisms (17). *P. gingivalis* has been shown to induce the expression of interleukins, tumor necrosis factor (TNF)-*α*, and matrix metalloproteinases (MMPs), as well as to inhibit apoptosis and suppress the activity of the tumor suppressor gene *p53*. *F. nucleatum* promotes cell proliferation and upregulates the production of interleukins and additional MMPs—components which, in conjunction with DNA mutations, may facilitate tumor invasion and metastasis, particularly in individuals with pre-existing potentially malignant disorders (18).

### 1.3 Antimicrobial peptides

**Figure 1:**
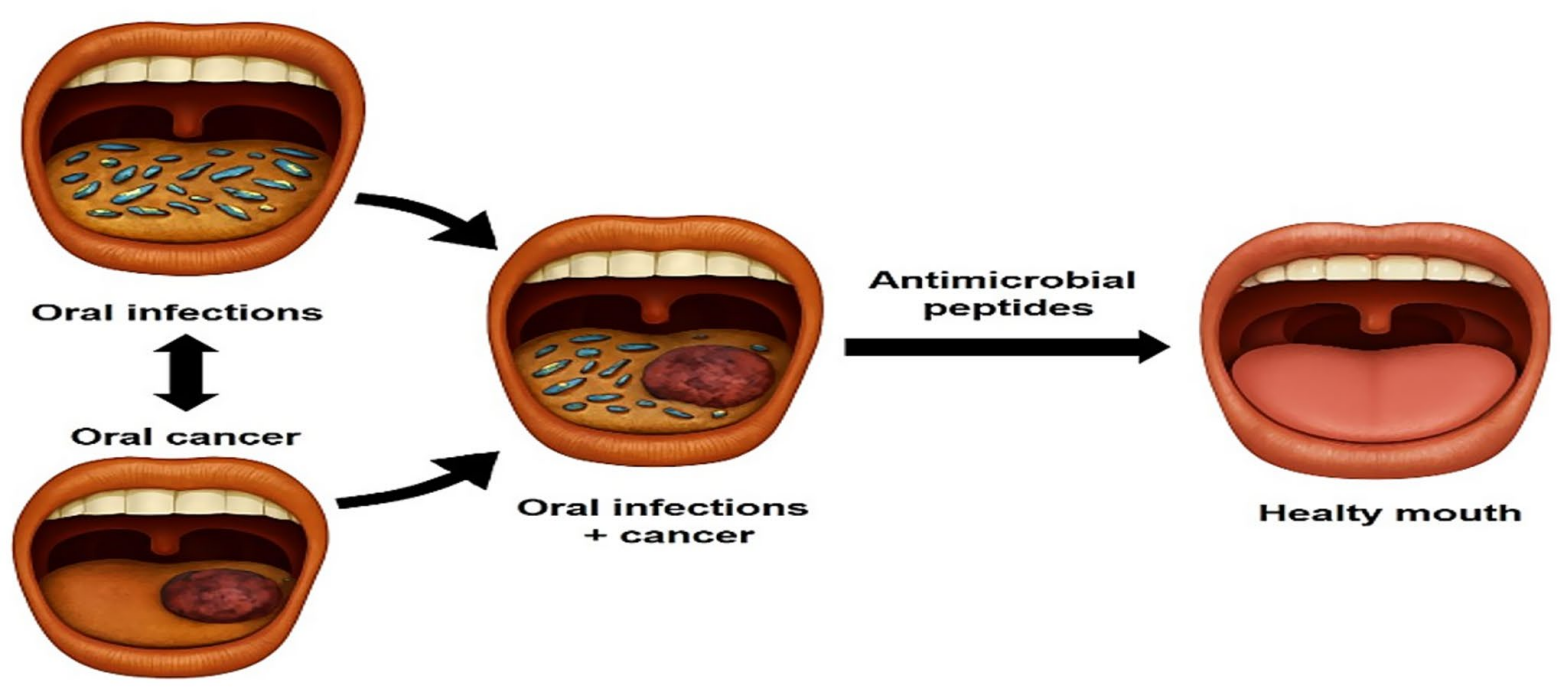
Antimicrobial peptides as potential molecules to treat oral infections and cancer.

To address this complex relationship between bacterial infections and oral cancer, antimicrobial peptides (AMPs) emerged as a promising approach (19). More than 100 peptide-based drugs have been approved by the Food and Drug Administration (FDA), and projections for the therapeutic peptide market indicate significant growth (20). Peptide molecules play a significant physiological and biochemical roles in many living organisms, exhibiting a wide range of functions such as neuromodulators, neurotransmitters, and hormones. Furthermore, peptides also play roles in metabolic, reproductive, and immune response processes (21). Among the diverse biological activities exhibited by peptides, their antimicrobial function is particularly noteworthy. AMPs are typically cationic polypeptides and exhibit antimicrobial activity against bacteria, fungi, parasites, and viruses (22). AMPs exert their antimicrobial effects by disrupting microbial membranes, interfering with intracellular processes, and modulating the host immune response (23). In addition to their antimicrobial properties, many AMPs exhibit anticancer activity (24). These peptides act via different mechanisms such as disruption of the cell membrane, DNA-related damages, inhibition of angiogenesis, regulation of immune cells/modulation of immune response, and interference with cell proliferation/cell cycle regulation (25, 26). The ability of certain AMPs to possess both antimicrobial and antitumor activities makes them particularly attractive candidates for the therapeutic approach to oral cancer and infections (Figure 1).

Therefore, this work aimed to find dual-function peptides with potential antimicrobial and antitumor activity against *P. gingivalis* and *F. nucleatum* as well as oral cancers. To achieve this goal, an initial literature search was conducted in PubMed by two independent reviewers using the following strategy: (“Antimicrobial peptides” OR “Antimicrobial peptide”) AND (“*Porphyromonas gingivalis*” OR “*Fusobacterium nucleatum*”). From the articles retrieved, peptides demonstrating antibacterial activity against both bacterial species were selected for further analysis. To evaluate the potential antitumor properties of these peptides, a second search was performed in PubMed using the terms: (“anti-cancer” OR “anticancer” OR “antitumor”) AND the specific name of each peptide found. Any discrepancies between the authors were resolved through discussion.

## 2 Discussion

Our initial literature search was conducted in order to identify AMPs with activity against *P. gingivalis* and/or *F. nucleatum*, the main oral pathogens implicated in carcinogenesis. Peptide name, sequence, and references are listed in Table 1.

**Table 1:** Antimicrobial peptides with proved activity against *P. gingivalis* or *F. nucleatum.*

Peptide	Source	Sequence	Activity	References
DP7	Designed	VQWRIRVAVIRK	*P. gingivalis*	(58)
Pep-7	Designed	RPHGAGEGIDRVPAGP-SPSEVGLAIPSGK	*P. gingivalis*	(59)
Amyl-1-18	Derived from α-amylase in rice	HLNKRVQRELIGWLDWLK	*P. gingivalis*	(60, 61)
Microcin C7	*E. coli*	MRTGNAD	*P. gingivalis*	(62, 63)
TPS-032	Putative peptide from *P. bremeri*	RVLTHVFKCKLKLR	*P. gingivalis*	(64)
Nisin z	*Lactococcus lactis*	ITSISLCTPGCKTGALMGCNMKTATCNCSIHVSK	*P. gingivalis* and *F. nucleatum*	(42)
K4-S4 (1-15)a	Tree frog skin	LWKTLLKKVLKAAA-NH_2_	*F. nucleatum*	(65)
Dhvar4a	Human	KRLFKKLLFSLRKY-NH_2_	*F. nucleatum*	(65)
SCPPPQ1	Human	FPLPPQPP	*P. gingivalis*	(66)
Br-J-I	Designed	PFaKLSLHL-NH_2_	*P. gingivalis*	(67)
LL37f	Human	KRIVQRIKDFLRNLVPRTES	*F. nucleatum*	(46)
KR12	Human	KRIVQRIKDFLR	*F. nucleatum*	(46)
LyeTxI	Venom of the spider *Lycosa erythrognatha*	IWLTALKFLGKNLGKHLAKQQLAKL	*P. gingivalis* and *F. nucleatum*	(46)
κ-casein (109–137)	Fragment of k-casin (bovine)	PPKKNQDKTEIPTINTIASGEPTSTPTTE	*P. gingivalis*	(68)
Nal-P-113	Designed	Ac-AKR-Nal-Nal-GYKRKF-Nal-NH_2_	*P. gingivalis* and *F. nucleatum*	(32, 34)
LL-31	Human	LLGDFFRKSKEKIGKEFKRIVQRIKDFLRNL	*P. gingivalis*	(69)
hCAP (109–135)	Human	FRKSKEKIGKEFKRIVQRIKDFLRNLV	*P. gingivalis*	(35)
Glycinin-17	Soybean	RKSREWRSKKTQPRRPR	*P. gingivalis*	(70)
BCAS-16	Soybean	KNQYGRIRVLQRFNQR	*P. gingivalis*	(70)
BCBS-11	Soybean	RIRLLQRFNKR	*P. gingivalis*	(70)
Plantaricin 149	*Lactobacillus plantarum*	YSLQMGATAIKQVKKLFKKKGG	*P. gingivalis*	(71)
Hsp-70 (241–258)	Fragment of heat shock protein (rice)	DNRMVNHFVQEFKRHKK	*P. gingivalis*	(72)
MUC7-12-mer-D	Designed	RKSYKCLHKRCR	*P. gingivalis*	(73)
CL (14–25)	Rice	RRLMAAKAESRK	*P. gingivalis*	(74, 75)
CL (K25A)	Rice	RRLMAAKAESRA	*P. gingivalis*	(75)
KR-12-a5	Human	KRIVKLILKWLR-NH_2_	*F. nucleatum*	(76)
PAC-525	Designed	Ac-KWRRWVRWI-NH_2_	*P. gingivalis* and *F. nucleatum*	(47)
KN-17	Designed	KWKVFKKIEKMGRNIRN	*F. nucleatum*	(77)
PGLa-AM1	Frog *Xenopus amieti*	GMASKAGSVLGKVAKVALKAAL-NH_2_	*F. nucleatum*	(78)
CPF-AM1	Frog *Xenopus amieti*	GLGSVLGKALKIG ANLL-NH_2_	*F. nucleatum*	(78)
Magainin-AM1	Frog *Xenopus amieti*	GIKEFAHSLGKFGKAFVGGILNQ	*F. nucleatum*	(78)
SMAP-28	Sheep	RGLRRLGRKIAHGVKKYGPTVLRIIRIA-NH_2_	*P. gingivalis* and *F. nucleatum*	(79, 80)
SMAP-29	Sheep	RGLRRLGRKIAHGVKKYGPTVLRIIRIAG	*P. gingivalis* and *F. nucleatum*	(80, 81)
WLBU2	Designed	RRWVRRVRRWVRRVVRVVRRWVRR	*P. gingivalis* and *F. nucleatum*	(82)
KSL	Designed	KKVVFKVKFK-NH_2_	*P. gingivalis* and *F. nucleatum*	(47, 48)

AMPs active against *P. gingivalis* or *F. nucleatum* ranged from natural to rationally designed, from short to long sequence, and from early 21st century to recent studies. The number of AMPs against these two bacteria appears to be large, but it is actually small when considering the thousands of peptides with antimicrobial activity reported in the literature and databases (27, 28). This demonstrates that many AMPs have not yet been tested for activity against bacteria that cause oral infections.

Peptides such as Nisin, LyeTxI, Nal-P-113, PAC-525, SMAP-28/29, WLBU2 and KSL deserve special attention due to their activity against both *P. gingivalis* and *F. nucleatum*. It is important to note that peptides that are active against only one of the bacteria do not mean that they are not active against the other, but rather that they have not yet been tested.

A second search was performed in literature in order to find the potential antitumor properties of peptides listed in Table 1. Just three peptides that are active against the main oral carcinogenic bacteria also have activity against oral cancer cell lines (Table 2). However, there are several AMPs against *P. gingivalis* and *F. nucleatum* whose antitumoral activity has not yet been tested. Even so, these AMPs can contribute to the prognosis of the disease even without having antitumor activity.

**Table 2:** AMPs against *P. gingivalis* and *F. nucleatum* that also have antitumoral activity.

Peptide	Antimicrobial activity (MIC)		Antitumoral activity		Reference
	*P. gingivalis*	*F. nucleatum*	IC50	Cell lines	
Nal-P-113	20–31 μg/mL	23–40 μg/mL	25.91–28.11 μM	OEMC and C9	(29, 33, 34)
hCAP_(109–135)_	1.9 μg/mL^#^	ND	40 μg/mL^&^	SAS-H1	(35, 36)
Nisin z	15–20 μg/mL	50 μg/mL	>100 μg/mL	UM-SCC-17B, UM-SCC-14A, HSC-3 and OSCC-3	(42, 43)

Oral squamous cell carcinoma cell line OEMC-1 (OEMC). Gingival epidermoid carcinoma (C9). Oral squamous cell carcinoma cell line SAS-H1 (SAS-H1). Human HNSCC cell lines, UM-SCC-17B (supraglottis/soft tissue-neck), UM-SCC-14A (floor of mouth), oral SCC cell line HSC-3 (tongue), and oral SCC cell line OSCC-3 (tongue). ^#^The authors determined HC₅₀ instead of MIC. ^&^88 ± 7.2% percent of cytotoxicity. ND, not determined.

One peptide that perfectly met the requirements proposed in our search is the Nal-P-113. Briefly, Nal-P-113 is a variant of the AMP P-113, in which the histidine residues at positions 4, 5, and 12 are replaced by *β*-naphthylalanine (29). Its antimicrobial activity against *P. gingivalis* (strain W83) was determined by Wang et al., who reported a minimal inhibitory concentration (MIC) and minimum bactericidal concentration (MBC) of 20 μg/mL and 160 μg/mL, respectively. Against *F. nucleatum* (strain ATCC25586), the peptide showed a MIC of 40 μg/mL and an MBC of 160 μg/mL. *In vivo* studies showed a protective effect against *P. gingivalis*-induced periodontitis in rats by reducing bacterial load and regulating IL-1β and TNF-*α* production (30). Added to this protective effect, Nal-P-113 can accelerate the proliferation of human immortalized oral epithelial cells and the repair of oral mucosal tissue damage (31). Interestingly, when encapsulated, Nal-P-113 also effectively inhibited the growth of *P. gingivalis* and *F. nucleatum*, with MICs of 31 μg/mL and 23 μg/mL, respectively (32). The antitumoral activity of Nal-P-113 was tested against the squamous cell carcinoma cell line OECM-1 and gingival epidermoid carcinoma cell line C9. The 50% inhibitory concentration (IC50) was 25.91 μM for OECM-1 and 28.11 μM for C9 (33). Yet another feature to mention is that Nal-P-113 showed safety and did not cause human periodontal ligament stem cells (hPDLCs) and human gingival epithelial cells (epi4) death at concentrations below 320 μg/mL (34). Taken together, the data highlight the broad potential of Nal-P-113 for clinical applications.

Another peptide that also met our research criteria is hCAP_(109–135)_, a 27-amino acid sequence of the C-terminal domain of the human-derived CAP18. hCAP_(109–135)_ presented an IC50 for *P. gingivalis* of 1.9 μg/mL (35). However, its activity against *F. nucleatum* has not been evaluated yet. With regard to the antitumoral activity, the peptide induced apoptosis in human oral squamous cell carcinoma, SAS-H1 cell, presenting 88 ± 7.2% percent of cytotoxicity at 40 μg/mL. Interestingly, the peptide was safe for healthy human gingival fibroblasts (36).

The C-terminal end of human CAP18 also contains a 37-amino acid peptide called LL-37, widely studied over the past years (37). LL-37 has been reported to be present in human saliva, gingival crevicular fluid, and gingival and buccal epithelium (38, 39). From the perspective of antimicrobial activity, LL-37 presented a MIC of 100 mg/L against *P. gingivalis* and 4.9 to 25 mg/L against *F. nucleatum* (40). The peptide also exhibits a dual role in cancer, displaying both anti-cancer and pro-cancer effects depending on the specific kind of cell (37). LL-37 has been shown to inhibit proliferation and induce autophagy and apoptotic cell death in oral squamous cell carcinoma. On the other hand, for cancers such as lung, breast, ovarian, melanoma, prostate, liver, and cutaneous squamous cell carcinoma, it appears to promote proliferation, migration, and tumor progression. However, the underlying mechanisms behind these contrasting roles of LL-37 remain unclear (41).

Finally, Nisin Z, a well-known peptide used as a food preservative, also matches our research criteria. Nisin Z possesses great potential for treating *P. gingivalis* and *F. nucleatum* with MIC values ranging from 15 to 50 μg/mL. Furthermore, the peptide interferes with biofilm development and reduces biofilm biomass of saliva-derived multispecies bacteria without inducing cytotoxic effects in human oral cells (42). Regarding antitumor activity, Nisin Z was active against different human head and neck squamous cell carcinomas, such as supraglottic/soft tissue-neck, floor of mouth, and tongue, by inducing apoptosis while suppressing proliferation, clonogenicity, and sphere formation. Importantly, Nisin Z activated a calpain-dependent apoptotic pathway selectively in HNSCC cells, without affecting normal human oral keratinocytes (43). Notably, Radaic et al. showed that solid lipid nanoparticles loaded with nisin can inhibit the growth of oral planktonic and biofilm pathogens and decrease OSCC viability compared to free nisin (44).

There are some other peptides with antitumoral activity against oral cell lines. A remarkable example of that is Pardaxin, a 33-amino-acid-residue peptide with activity against oral squamous carcinoma (45). Interestingly, pardaxin has not been tested against *P. gingivalis* or *F. nucleatum*, presenting a promising opportunity for researchers. Like pardaxin, other peptides with proven antitumor activity have not yet been evaluated against potentially oral carcinogenic pathogens and are therefore great promising molecules in the fight against oral cancer and infections. It seems that many research groups work specifically on evaluating peptides as potential antibiotics, while others work on evaluating antitumor activity. Many of these molecules do not have their potential dual activity effectively evaluated, thus being underestimated. This scenario is highlighted for the case of oral cancer, for which few AMPs have been evaluated. Specifically, peptides like KSL and Lye-Tx-I exhibit both antimicrobial and antitumoral activities (46–51). However, their antitumoral effects have been demonstrated against other cancer types—such as colorectal adenocarcinoma and breast cancer cell lines—with no reported efficacy against oral cancer (49–51).

Even though it is not the focus of this work, it is important to mention that besides bacteria, other pathogens such as viruses and parasites are also considered tumorigenic (52–54). In this regard, there are AMPs that are active against these pathogens, expanding the potential of these molecules for cancer prevention/treatment (55, 56). However, due to the limitations of AMPs, further studies are needed before their commercialization as therapeutic agents for the treatment of oral infections and cancer. For instance, most AMPs are susceptible to proteases and have limited activity at physiological conditions, leading to a reduced *in vivo* effectiveness. Also, toxicity to host cells and cost of production are still the main challenges that AMPs have to face. Fortunately, there are several strategies that researchers can use to minimize or even overcome those problems, such as improved production strategies, peptide activity optimization via artificial intelligence, and delivery strategy optimization (23, 57). Moreover, combining AMPs with conventional treatments like chemotherapy and radiotherapy may improve therapeutic effectiveness and minimize resistance, offering more effective strategies for managing oral cancer.

## 3 Conclusion

Even though there are many review articles on antimicrobial peptides with antitumor activity, our work can be considered pioneering. We compile promissory AMPs to treat both oral cancer and infections. AMPs such as Nal-P113, hCAP_(109–135)_ and Nisin Z could potentially combat infections caused by *P. gingivalis* and *F. nucleatum*, which are implicated in oral tumor development, having also direct antitumoral activity. Furthermore, we list several other peptides that do not have their potential dual activity evaluated to date. Our review encourages researchers to continue exploding the potential of AMPs and face the challenges that hinder their clinical application for cancer.
